# 2305: Advancement of translational sciences: Development of an interprofessional program and outcome measures for foundational, clinical, and health care researchers

**DOI:** 10.1017/cts.2017.171

**Published:** 2018-05-10

**Authors:** Gayathri Devi, Ranjan Sudan, Stephanie Freel, Laura Fish

**Affiliations:** Duke University, Durham, NC, USA

## Abstract

OBJECTIVES/SPECIFIC AIMS: To improve translational research, we have developed a program called Duke Multidisciplinary Education and Research in Translational Sciences (Duke MERITS). Duke MERITS will facilitate cross-disciplinary collaboration among faculty involved in foundational, clinical and/or health care research and in turn also prepare them to train the next generation of translational researchers. METHODS/STUDY POPULATION: The program aims are (1) to define metrics and outcomes measures so faculty can track their progress and identify impact of their collaborative research in translational sciences; (2) to offer a multi-modal faculty development series to promote team science, improve didactic teaching, and incorporate innovative resources to promote interdisciplinary approach to translational research; (3) to provide module-based hands-on-training sessions in bench to bedside research and training in translational grant writing to facilitate the development of multidisciplinary research collaborations. The present study describes results from Aim 1 and includes (a) development of baseline outcome assessment tools necessary to gauge the impact of our programs on both the participating faculty and the research culture within Duke University, (b) impact of a specific course offering in Translational Medicine. In order to achieve this, we conducted multiple focus group sessions with faculty self-identified as junior-, mid-, or advanced-career, a mixed group at any career level and included a group of graduate students and postdoctoral trainees to study the impact of a graduate level course in Translational Aspects of Pathobiology. The activities during these translational science focus groups were designed to define what successful translational science is, to determine what resources support translational Science at Duke, and to decide what resources we need in order to enhance Duke’s position as a leader in research and scientific education. RESULTS/ANTICIPATED RESULTS: We identified that translational science is changing standards while incorporating leadership, teamwork, collaborations, and movement primarily focusing on the overall goal of improving all aspects of health. Participants categorized their field of study and the fields of their coparticipants most frequently as basic discovery and a combination of intervention and health services. The most frequently identified pros/benefits of performing translational science at Duke include industry connections, collaborations with other departments resulting in disciplines being bridged, improving patient care, and access to resources as well as money. The most frequently identified cons/barriers of performing translational science includes the expensiveness, silos, and lack of resources willing to absorb risks. DISCUSSION/SIGNIFICANCE OF IMPACT: The identification of these defined factors from the focus groups has allowed us to issue a comprehensive, sliding Likert scale-based anonymous survey from the secure RedCap system and is being rolled out throughout Duke University, including schools of medicine, nursing, Trinity, biomedical engineering. We envision that Duke MERITS education program will facilitate interprofessional efforts, which we define as a team science approach to identify the clinical “roadblock” and then seek an innovative approach or technology to help overcome this “roadblock”? It can facilitate institutional and departmental recognition in faculty career development. The common goal is to gain fundamental new insights that will result in significant improvement of the existing “standard of care” and meet the challenges of dwindling extramural support.

